# Clinicians’ experience of barriers and facilitators to care delivery of an extracorporeal cardiopulmonary resuscitation service for out-of-hospital cardiac arrest: a qualitative survey

**DOI:** 10.1186/s13049-024-01261-7

**Published:** 2024-09-13

**Authors:** Jasper Eddison, Oscar Millerchip, Alex Rosenberg, Asher Lewinsohn, James Raitt

**Affiliations:** 1 National Heart and Lung Institute, Imperial College London, London, UK; 2 Harefield Hospital, Harefield, UK; 3 Thames Valley Air Ambulance, Stokenchurch, UK

**Keywords:** Extracorporeal cardiopulmonary resuscitation (ECPR), Out-of-hospital cardiac arrest (OHCA), Qualitative analysis, Clinician perspective

## Abstract

**Background:**

Out-of-hospital cardiac arrest (OHCA) survival in the UK remains overall poor with fewer than 10% of patients surviving to hospital discharge. Extracorporeal cardiopulmonary resuscitation (ECPR) is a developing therapy option that can improve survival in select patients if treatment begins within an hour. Clinicians' perspectives are a pivotal consideration to the development of effective systems for OHCA ECPR, but they have been infrequently explored. This study investigates clinicians' views on the barriers and facilitators to establishing effective systems to facilitate transport of OHCA patients for in-hospital ECPR.

**Methods:**

In January 2023, Thames Valley Air Ambulance (TVAA) and Harefield Hospital developed an ECPR partnership pathway for conveyance of OHCA patients for in-hospital ECPR. The authors of this study conducted a survey of clinicians across both services looking to identify clear barriers and positive contributors to the effective implementation of the programme. The survey included questions about technical and non-technical barriers and facilitators, with free-text responses analysed thematically.

**Results:**

Responses were received from 14 pre-hospital TVAA critical care and 9 in-hospital clinicians’ representative of various roles and experiences. Data analysis revealed 10 key themes and 19 subthemes. The interconnected themes, identified by pre-hospital TVAA critical care clinicians as important barriers or facilitators in this ECPR system included educational programmes; collectiveness in effort and culture; teamwork; inter-service communication; concurrent activity; and clarity of procedures. Themes from in-hospital clinicians’ responses were distilled into key considerations focusing on learning and marginal gains, standardising and simplifying protocols, training and simulation; and nurturing effective teams.

**Conclusion:**

This study identified several clear themes and subthemes from clinical experience that should be considered when developing and modelling an ECPR system for OHCA. These insights may inform future development of ECPR programmes for OHCA in other centres. Key recommendations identified include prioritising education and training (including regular simulations), standardising a ‘pitstop style’ handover process, establishing clear roles during the cannulation process and developing standardised protocols and selection criteria. This study also provides insight into the feasibility of using pre-hospital critical care teams for intra-arrest patient retrieval in the pre-hospital arena.

**Supplementary Information:**

The online version contains supplementary material available at 10.1186/s13049-024-01261-7.

## Background

OHCA is common with an estimated 30,000 resuscitation attempts in England per year alone [1], but survival to discharge remains relatively poor at less than 10% [1–4]. OHCA care depends on the chain of survival, which includes an early call for help, prompt cardiopulmonary resuscitation (CPR), defibrillation, and post-resuscitation care [5–7]. Increasing the rates of bystander CPR and early defibrillation can increase survival rates to as high as 25% [8], and thus is already an identified area of priority [6, 9, 10].

When CPR and defibrillation attempts are unsuccessful, some patients may benefit from extracorporeal cardiopulmonary resuscitation (ECPR). ECPR involves the rapid initiation of veno-arterial extracorporeal membrane oxygenation (ECMO) during continued CPR efforts [11]. Its goal is to artificially restore adequate tissue perfusion and oxygenation to prevent multi-organ failure and hypoxic brain injury [12–14]. ECPR effectively prolongs the possible survival time window during resuscitation, allowing time for diagnostic and therapeutic interventions [15, 16], primarily percutaneous coronary intervention, in order to address the underlying pathology and cause [17].

Recent studies on the efficacy of ECPR have shown some encouraging results but other studies with mixed results are a reminder of the complexity of the logistics and system design required. One observational study demonstrated potentially a 54% survival rate in patients with refractory cardiac arrest [18], though only one of three subsequent randomised controlled trials demonstrated a significant improvement [19–21]. These findings indicate that ECPR can work but there remains a high variability between centres and multiple confounding factors that need further research and consideration [22]. Providing an effective ECPR service is complex and compounded by its high-acuity, low-occurrence (HALO) nature [23]. It requires a multidisciplinary and multi-service collaboration adapted to distinct regional needs and pre-existing health systems [14, 24, 25]. There is a general recognition that any aspect of cardiac arrest care in isolation is of little value and that it takes an integrated system to save a life [6, 19, 26, 27].

Studies have consistently demonstrated that the shorter the duration from collapse to initiation of ECPR, the better the rates of survival and neurological outcomes [28–31]. ECPR success therefore depends on a careful balance between early patient identification, speed of decision making and timely move to intervention. For every minute delay initiating ECPR after cardiac arrest, there is a significant impact on the chances of survival [32–34], and when this interval exceeds 60 min, overall long-term survival outcomes are universally poor [14]. Appropriate patient selection is equally as important as speed of initiation, which must be carefully considered to ensure that the underlying pathology and patient physiology are compatible with a long-term positive prognosis. This is to balance between the increased costs and risks of the procedure vs the benefit gained for patient care. Ideal patient characteristics identified for optimal outcomes include patients who present in an initially shockable rhythm [31, 35], have a witnessed collapse with effective immediate bystander CPR, as well as a lower perceived clinical frailty [33]. Importantly, system enhancements to improve patient selection [36, 37] and minimise low-flow (chest compression) duration [22, 38] have been shown to improve ECPR outcomes.

Previous research has highlighted other potential barriers to achieving these aspirations including geographical and infrastructural challenges in accessing the specialist care needed in a timely fashion [39, 40]. Similarly, challenges in the clinical ability to rapidly assess individual patient eligibility for this invasive procedure whilst still providing effective resuscitation and patient care have been documented and explored [41, 42]. Further barriers also recognised include the perceived lack of effectiveness by clinicians of the procedure vs the high cost and staffing implications [43], and time delaying factors during the technical canulation process [44].

However, the literature lacks an exploration of clinicians' perspectives on some of these challenges and how these systems operate. Previous research has identified the need for qualitative analysis of factors that enhance or hinder systems [42]. Furthermore, few studies and guidelines provide practical recommendations for effective pre-hospital systems that are generalisable to a UK setting [14].

TVAA started air operations in the Thames Valley region in 1999 and now operates in partnership with South Central Ambulance Service (SCAS) as the primary regional provider for advanced pre-hospital critical care services between the hours of 0700–0200, 365 days-a-year. The service model aims to dispatch critical care doctors and paramedics on both a car based and EC135 helicopter-based platform to scene alongside the ambulance service within the mixed urban and rural Buckinghamshire, Berkshire and Oxfordshire areas they serve. 28% of the caseload attended by the service are OHCA’s, although only patients within a 30-min drive to Harefield Hospital were considered for inclusion to the ‘ECPR project’ due to logistical, safety and time constraints. Harefield Hospital is a specialist cardiothoracic centre of excellence located in North-West London, providing primary cardiothoracic, angioplasty and mechanical circulatory support services [45].

**Fig. 1:**
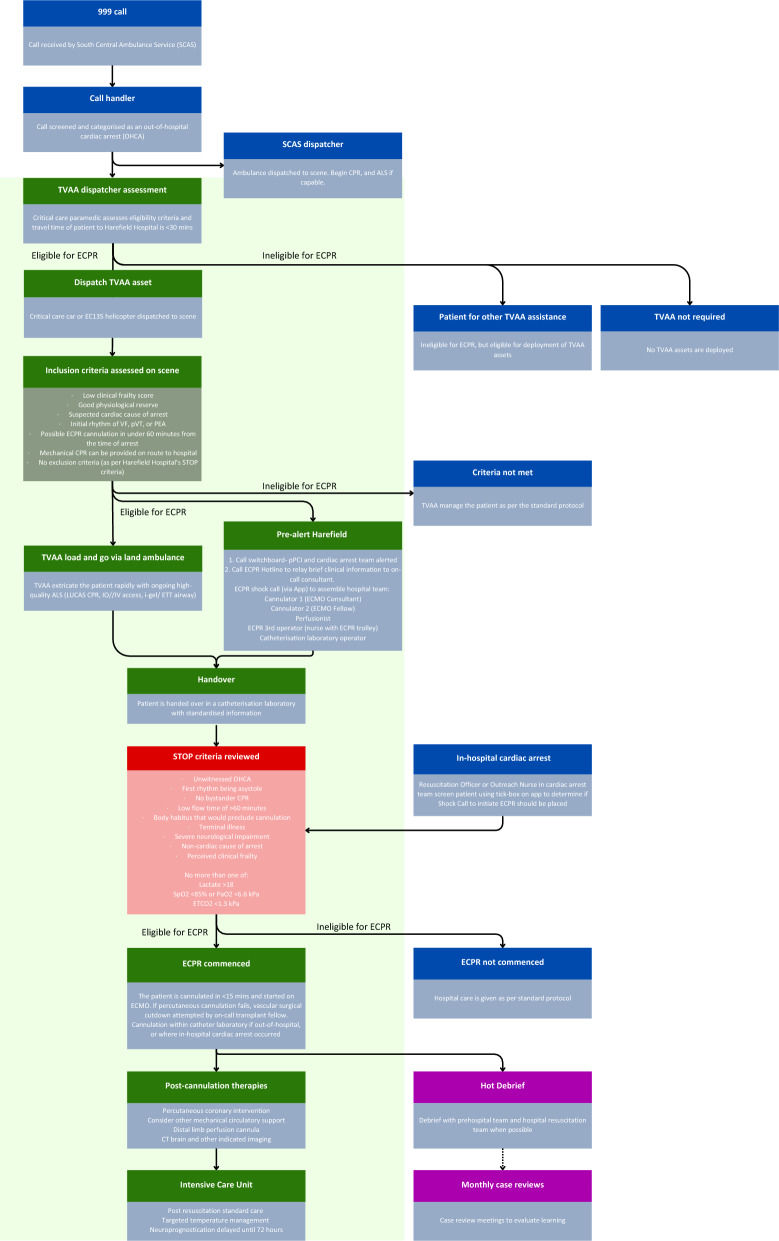
Flow diagram depicting key steps of the ECPR protocol. Light green depicts ECPR protocol continuation. TVAA, Thames Valley Air Ambulance. SCAS, South Central Ambulance Service. ECPR, extracorporeal cardiopulmonary resuscitation. ECMO, extracorporeal membrane oxygenation. Created with Canva.com

In January 2023, Thames Valley Air Ambulance (TVAA) and Harefield Hospital established the first formal system in the UK for selecting patients in refractory OHCA to receive a fast-track process for intra-arrest transfer to an ECMO centre for ECPR. This system differs from previously established rendezvous [46] and out-of-hospital cannulation systems [47], and utilises specialist critical care pre-hospital teams instead of road SCAS ambulance service teams for patient selection and transfer. This aimed to maximise the ability and confidence of clinicians to provide rapid patient identification, selection, transfer, and retrieval whilst maintaining high-quality resuscitation efforts on-route. Figure 1 outlines the system design and protocols that were eventually developed and agreed upon for use in this system design.

This study aimed to evaluate clinicians' perspectives on the barriers and facilitators to care delivery in this unique system that aims to provide joined-up care between pre-hospital TVAA critical care and in-hospital teams for the delivery of ECPR for refractory OHCA. While this system has been developed specifically for the local region, we hope this study will help facilitate a better understanding of the process and challenges involved in setting up a process like this and in doing so, help other healthcare services aiming to establish similar systems to improve equitable cardiac arrest care across the UK.

## Methods

### Design

This study used an online, single-centre, cross-sectional survey which was chosen as an effective means of capturing a wide range of perspectives in an unexplored area [48]. The survey is reported according to Checklist for reporting results of internet E-surveys (CHERRIES) [49], and qualitative reporting according to Standards for Reporting Qualitative Research (SRQR) [50].

The survey questions probed for technical and non-technical barriers and facilitators in the cases in which participants had been involved, and one further question asked participants to provide advice to their counterparts in another service. The research team opted for five mandatory free-text questions to allow participants to identify and formulate ideas themselves. Participants were able to review and change answers until submission.

The survey conducted was anonymous and consisted of a participant information sheet that outlined the survey’s aims, length, data handling and who the investigatory team were, before a mandatory consent checkbox to proceed. Eight clinicians and non-clinicians piloted the survey for content accuracy and technical functionality.

### Population and recruitment

Pre-hospital TVAA critical care teams (doctors and paramedics); Harefield hospital cannulating doctors, nurses and perfusionists; and ECPR-experienced intensive care clinicians were eligible to participate. The survey was sent to team members involved in ECPR pathway activation, regardless of outcome or cannulation. Additionally, clinical leads who helped establish the service were included to provide a strategic-level perspective.

The TVAA research lead and ECMO lead at Harefield Hospital identified and distributed the open survey link to eligible participants via institutional email with follow-up emails and regular informal reminders. There was no incentive, and the survey was completely voluntary. Snowball sampling was allowed and encouraged for study participants who may not have originally been identified (for example, clinicians who may have joined the department at a later date or been cross-covering for regular staff involved in the programme for certain cases) and therefore a final sample size was difficult to identify especially for in-hospital teams. This is a recognised limitation of this study.

### Data collection

Data collection occurred between 6th March and 27th April 2024. Qualtrics XM was used to securely host the online survey [51]. Completed surveys were exported to an encrypted Microsoft Excel document, with any included email addresses being removed to fully anonymise responses before analysis.

### Data analysis

All submitted free-text responses were analysed using thematic analysis following the framework from Braun and Clarke, focusing on a semantic description of the whole dataset with an inductive approach with deductive aspects [52]. NVivo 14 software was used to organise and manage the data during the qualitative analysis [53]. The analytical process included familiarisation of the dataset, identification of codes, collating codes into distinct categories, and refinement of overarching themes and subthemes. Pre-hospital TVAA critical care and hospital responses were analysed separately,however, all free-text responses were analysed collectively regardless of the prompt question. To increase the trustworthiness of the findings, a second appraiser independently reviewed the full dataset, and new or contrived themes and categories were discussed until a consensus was reached.

### Ethical approval

Using the Health Research Association decision tool, formal NHS Research Ethical Committee approval was deemed not necessary [54]. Institutional approval from TVAA (Ref-AIR/01/24) and Harefield Hospital (reviewed by lead for clinical risk) was granted. No patient-identifiable data were collected at any point.

## Results

The response rate for TVAA clinicians was 14/17 (82%). Nine in-hospital clinicians responded, however the survey denominator for the in-hospital team was unknown. Participants’ background and information is displayed in Table 1, showing representation of varied roles and experience.

**Table 1:** Background characteristics of participants

Characteristic	Number (%)
TVAA	14 (61)
Doctor	9 (64)
Critical care paramedic	5 (36)
Number of OHCA cases	
0	1 (7)
1	11 (79)
2	2 (14)
> 2	0 (0)
Harefield Hospital	9 (39)
Doctor	3 (33)
Intensive care	2 (22)
Cardiology	1 (11)
Nurse	4 (44)
Intensive care	3 (33)
Unknown	1 (11)
Perfusionist	2 (22)
Number of OHCA ECPR cases	
0	2 (22)
1–5	0 (0)
> 5	7 (77)
Number IHCA ECPR cases	
0	0 (0)
1–5	2 (22)
6–10	1 (11)
11–15	0 (0)
> 15	6 (66)

Percentages of TVAA and Harefield Hospital of total respondents (/23). All other percentages describe the proportion of TVAA (/14) or Harefield Hospital (/9) clinicians

*TVAA* Thames Valley Air Ambulance. *OHCA* out-of-hospital cardiac arrest. *IHCA* in-hospital cardiac arrest. *ECPR* extracorporeal cardiopulmonary resuscitation

**Fig. 2:**
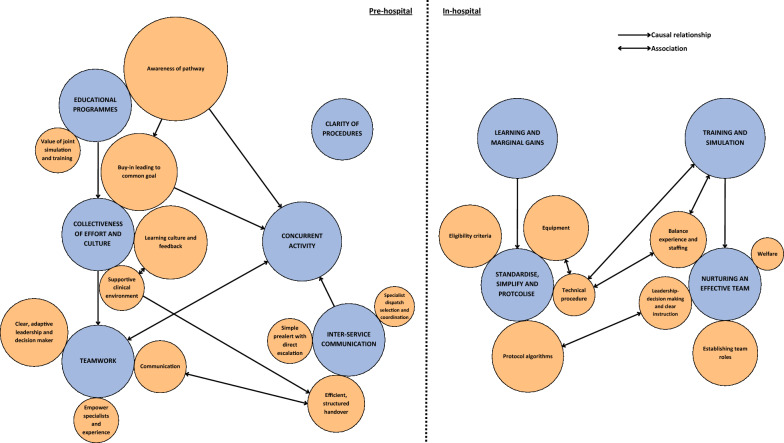
Graphic representation of the relationships between themes (blue) and sub-themes (orange). The size of the bubble represents the number of codes. Created with Canva.com

The findings presented in this section attempt to address the research aim in question by identifying barriers and facilitators to the development of an ECPR pathway for OHCA. Analysis of the data collected identified 10 key significant themes. Some themes were standalone, while others were constituted of subthemes, of which there were 19. These are reported in two sections: pre-hospital and in-hospital. Illustrative example comments for each theme and subtheme are presented in Table 2 and in the narrative below, shown with a responder number (pre-hospital: R1-14, in-hospital: R15-24). A visual representation of the relationships is displayed in Fig. 2.

**Table 2:** Results of thematic analysis themes and subthemes with illustrative quotes

Theme	Subtheme	*Illustrative examples (direct quotes)*	Coding frequency	Individual frequency
*Pre-hospital: TVAA critical care teams*				(/14)
Educational programmes	Awareness of pathway	*We anticipated that our teams might meet resistance to the concept of load and go from ambulance service staff, when for years they have been taught to stay at the scene until ROSC [return of spontaneous circulation] is established. Our anticipation of this challenge was correct as this has been the largest barrier, the situation has improved as the pathway becomes better established and additional briefings to our partner services have occurred. (R12)* *You'll need a big education program for the local ambulance service explaining why select patients will be 'scooped and resuscitated en route' because with the expansion of critical care / HEMS [Helicopter Emergency Medicine Services] teams working prehospitally, the norm has become to 'stay and play' for cardiac arrest, moving only if ROSC achieved and once patient is stabilised. (R1)*	27	12
	Value of joint simulation and training	*I have been involved with the ECPR training between TVAA & Harefield and have found this to have been invaluable when dealing with these cases (R4)*	9	5
Collectiveness in effort and culture	Buy-in leading to a common goal	*We were able to announce to the pre-hospital teams that we needed the patient on the ambulance in this time frame. This focussed efforts on a goal and the pre-hospital teams worked well together in order to achieve this. (R7)* *Local SCAS [South Central Ambulance Service] paramedics were aware of […] offering ECPR to certain patient groups and were aligned with the need to prioritise leaving scene (as opposed to completing ALS interventions, as per most cardiac arrests attended). This was important, as without this immediate buy-in, we could easily have been delayed on scene. (R14)*	17	11
	Learning culture and feedback	*Regular shared governance and review of all cases, our reviews included joint simulations. (R12)*	16	8
	Supportive clinical environment	*There is also a warm, sincere 'welfare check' on the crews after which is appreciated, as it is often quite a 'tour de force' to get these patients to Harefield within the required time frame. (R1)*	9	5
Teamwork	Clear, adaptive leadership and decision maker	*Crews were happy for me to take the lead in decision making and followed instructions well. (R3)*	11	7
	Communication	*Good communication when arrived at Harefield—receiving team listened to handover and then immediately commenced respective roles, which made this phase succinct. (R3)*	8	8
	Empower specialists and experience	*Being empowered to do the minimum necessary intervention at scene and en route to hospital to minimise on scene time. (R7)* *Various approaches have been taken regarding airway management including transfer to ECPR on an iGel, intubation on route and pausing transfer briefly to intubate. All these are reasonable approaches and allowing clinicians to exercise their judgement is reasonable. (R12)*	9	5
Interagency communication	Specialist dispatch selection and coordination	*Support from HEMS desk paramedic to calculate estimated timings (time since arrest, anticipated time to Harefield Hospital and hence time limitation on scene) (R14)* *HEMS desks/control room dispatchers’ communication with attending crews that the patient maybe a candidate for ECPR […] This will serve the attending crews/teams a reminder of the potential for patient treatment plan (R8)*	6	4
	Simple pre-alert with direct escalation	*The phone-call literally being a trigger to Switchboard rather than a clinical discussion- this is a huge strength of the Harefield pathway and a huge frustration in other pre-hospital (and in-hospital) pathways. (R7)*	9	6
	Efficient, structured handover	*Good communication when arrived at Harefield—receiving team listened to handover and then immediately commenced respective roles, which made this phase succinct. (R3)*	13	8
Concurrent activity		*[Ambulance] Crews and team leader were on board with the process and had been moving the scene on—Getting the scoop stretcher/stretcher ready and had an extrication plan (R8)* *Moving the case forwards and proceeding to ECMO until this decision to stop is made seems necessary to me if being timed out is a risk, as opposed to waiting until a definite yes before proceeding. (R9)* *Using time on the way to scene to calculate the time frames to successfully get the patient to Harefield within 1 h of cardiac arrest (R7)*	11	7
Clarity of procedures		*The instructions on the TVAA guidelines are clear and easy to follow. (R10)*	8	6
*In hospital: Harefield*				(/9)
Learning and marginal gains		*Debrief after complex cases to be able to learn (R17)* *We have conducted a major overhaul of the entire system from the bottom up and reviewed every element of the pathway looking to refine the technical elements of the procedure and improve speed, fluency and time. (R19)*	14	5
Standardise, simplify and protocolise	Protocol algorithms	*Having a standardized approach to every patient has been key within the ECPR service. (R21)* *Following protocols and shock protocols as listed in the trolley (R16)*	17	5
	Equipment	*Simplify equipment. We reduced multiple aspects of the procedure to the bare minimum (but still safe) number of steps. This included using less wires and less dilators, this reduced the time by over five minutes. Organise equipment—we packaged our equipment in a trolley that we can take anywhere in the hospital and open rapidly—minimising preparation time. […] we are currently commissioning a company to create an ECPR pack with our entire set of equipment contained within in it—saves time on opening and reduces errors. (R19)*	16	4
	Technical procedure	*We trained to use surface ultrasound techniques so that the imaging could be performed by the cannulators during the procedure without having to move the patient to fluro or relying on someone else to do a TOE [transoesophageal echocardiogram]. (R19)* *The nurses drill their steps to fit in with the natural longer parts of the procedure (so cannulators start prepping and draping, nurses prep sheaths needles and initial wires. During access they prep the stiff wires and dilators and try to start with the cannulae. While the cannulae are being inserted they prep the circuit so it's ready to be connected immediately—this saves many minutes from the procedure. (R19)*	8	3
	Eligibility criteria	*Having clear inclusion/exclusion criteria for decision making (R17)* *A major problem has been the paradigm shift in decision making. Hospital doctors like to make high quality, shared, well considered decisions involving data and an MDT [multidisciplinary team] of experts. The paradigm is right patient, right place, right time, right treatment by the right people. In ECPR this noble philosophy results in delay and either dead or brain damaged patients. Tick box screening and rapid / abbreviated ECMO cannulation appears to completely ignore this whole paradigm and makes clinicians extremely uncomfortable. (R19)*	14	5
Training and simulation		*All people present should have had ECPR training/simulation or be familiar with ECPR—to ensure shocks are stopped, compressions continued *etc.*… (R17)* *Simulation as much as possible between prehospital and in hospital team. (R21)*	14	5
Nurturing effective team	Leadership- decision making and clear instruction	*gave clear and precise instructions (R16)* *consultant led decision to progress with ECMO cannulation with clear guidance, helps reducing the risk of non-technical aspects affecting the procedure. (R21)*	11	8
	Establishing team roles	*Ensuring that everyone in the team knows their role and limitations. (R15)* *Different part of the teams dealt with different parts of the arrest, therefore that allowed minimal interruptions (R16)*	16	8
	Balance experience and staffing	*When there is a group of experienced staff it’s very straightforward (R20)* *Having a small team of cannulators, perfusion team and operators also helps to standardize the procedure and ensure the skills and confidence to perform ECPR. (R21)*	13	6
	Welfare	*There is an emotional impact to staff, psychology support must be available as well as a private space to decompress after withdrawal of therapy before resuming duties in theatre (R22)* *A hot debrief is valuable (R22)*	7	3

The number of extracts coded and the number of individual responders who had a comment coded is displayed

*HEMS* helicopter emergency medical services. *ECPR* extracorporeal cardiopulmonary resuscitation. *ECMO* extracorporeal cardiopulmonary resuscitation. *TVAA* Thames Valley Air Ambulance. *SCAS* South Central Ambulance Service

### Pre-hospital

#### Educational programmes

##### Awareness of pathway

The most common perceived barrier to reducing on-scene times was found to be the sub-theme 'awareness of pathway'. It was also identified as a key consideration for fostering better on-scene team dynamics and allowing shared mental modelling to facilitate a rapid change in team strategy from prior planned on-scene care to a rapid ‘load and go’ approach when the TVAA teams arrived. Effective dissemination of the new protocol across regional ambulance personnel to raise awareness of the new pathway was an anticipated challenge, which indeed materialised on several occasions. A lack of protocol awareness was identified in several cases as a root cause for delays on scene and friction between the working relationships of pre-hospital care teams.

*Ambulance crews who are uninformed as to the EPCR project can feel unhappy as to the nature of us ‘swooping’ and retrieving arrest pts they have been working on. (R4)*

In contrast, when all staff were informed of the new protocol, they were quick to adapt their practice:

*Crews and team leader were on board with the process and had been moving the scene on. (R8)*

##### Value of joint simulations and training

Respondents noted the value of joint training exercises in system implementation. TVAA and Harefield Hospital run regular simulations, often adapted to address any problems raised in shared clinical reviews*.* Clinicians appreciated the improved collaboration and efficiency between stages of the patient journey, with many suggesting further inter-service simulations with the ambulance crews:

*It is obvious we have collaborated and done joint simulations as it all just seems to flow naturally when we arrive. (R1)*

#### Collectiveness in effort and culture

##### Buy-in leading to a common goal

Clinicians perceived buy-in to the service as an essential attribute of all individuals involved, which was reliant on effective education about the reasons for ECPR. When all individuals understood the potential impact on the patient’s outcomes, a common goal was established that fostered efficient teamwork:

*We worked really well as a team; we all had the same goal and as a critical care team we were only on scene for 3 min. (R6)*

In one case, this common goal was suggested as counterproductive: “*There was definitely a hectic air on scene when people realised, we were trying to leave ASAP.” (R9)* However, this comment may be explained by a lack of preparedness and familiarity of the initial on-scene ambulance crew who may not have been briefed fully around what to expect, and how the scene dynamics may change, once an appropriate patient was identified by the critical care teams.

##### Learning culture and feedback

It was evident that the system had a strong learning culture with the opportunity to feedback at various debriefs and inclusive inter-service case reviews. It was apparent that each case, regardless of the outcome, was viewed as an opportunity to learn and improve rather than a failure:

*It was a very useful experience even though our patient didn't get cannulated. (R6)*

Multiple responses identified suggestions specific to this local system including protocol design, signage, navigation faults (“*ambulance was taken the wrong way to the hospital” (R8)*) and lapses in knowledge. These responses further highlight the opportunity to refine the system.

##### Supportive clinical environment

This sub-theme alluded to a system-wide culture of being friendly, supportive, and non-judgmental. This was most notably reported during the handover. The reception at the hospital was perceived to appreciate the concerted effort prior to arrival:

*Harefield always has a warm, non-judgmental reception. (R1)*

#### Teamwork

##### Clear, adaptive leadership and decision-maker

Having a clear leader who makes and communicates decisions effectively was considered important to gatekeep and drive progression. This leadership was required on-scene and upon arrival at the hospital. Leadership style, however, was context-dependent- a dynamic role requiring adaptation. In situations where everyone was aware of the process, assertive decisions were welcomed:

*Local ambulance service aware about ECPR pathway and receptive to decision making when I informed them we were attending Harefield.* (R3).

In contrast, announcing a decision could be perceived as taking over and pressurising: “*driven the scene too hard.” (R1).*

The comments above clarify the need for a leader to sensitively establish protocol awareness and a common goal.

##### Communication

Good communication within and between teams contributed to the establishment of a common goal on the scene, expressing a decision and ensuring clear roles were recognised. Factors such as time pressure and unfamiliarity with teams could undermine communication, whereas a supportive and professional environment facilitated effective communication:

*Communication ensured everyone knew what was required and potential clinical course for the patient. (R8)*

##### Empower specialists and experience

TVAA is a small, but high-performing cohesive team of specialists in critical care. Empowering practitioners to make clinical decisions in a dynamic situation and utilise skills acquired and maintained by routinely attending critically ill cases was perceived as valuable:

*Important to remain dynamic in decision making throughout case and respond appropriately to changing clinical picture. (R3)*

The technical competency and high resuscitation standard of the clinicians were noted:

*delivered high-quality CPR to the cases that we have transferred and this has been supported by feedback from the Harefield team. (R12).*

#### Inter-service communication

##### Specialist dispatch selection and coordination

Emergency calls are screened by experienced TVAA critical care paramedics from the ambulance operation centre. Their experience allowed them to use clinical judgment to identify potential patients with incomplete information; assist the crew with logistics such as calculating times; and advise ambulance crews on how to prepare:

*Crew had been made aware by our HEMS desk that ECPR was a possibility and had been given advice on steps to make the process smoother/quicker prior to our arrival. (R8)*

##### Simple pre-alert with direct escalation

The ease and effectiveness of pre-alerting were noted. The pre-alert involves a call which automatically triggers hospital escalation to prepare. An additional call to the on-call consultant was incorporated into the system to relay some clinical information to assist in the decision to open sterile equipment. Responders appreciated this allowed them to concentrate on clinical care: “*Helpful to place a single call *via* switchboard with minimal detail.” (R7).*

##### Efficient, structured handover

Pre-hospital TVAA critical care clinicians commented that a standardised, single handover to a ready, engaged hospital team improved efficiency: “*Good clear handover of care and quick decision to cannulate.” (R14).*

#### Concurrent activity

One theme defined was ‘concurrent activity’ which captured the parallel tasks occurring at a system and individual level. Time is the scarcest resource in this process, and utilising immutable periods (often transport) to progress the case was important to reduce scene time. This included ambulance personnel proactively preparing the patient for TVAA extraction, and interventions being initiated after departure:

*The patient was already being managed by [South Central Ambulance Service] […] saving time for when we arrived. (R8)*

#### Clarity of procedures

According to clinicians, having a clear and easily applied standard operating procedure and inclusion/exclusion criteria were helpful. These simplified a complex and infrequent operation to make them easier to remember or refresh on route to a case, and reduce the cognitive load required for a decision:

*Criteria to identify suitable candidate is clear and easy to apply. (R11)*

In contrast, when the protocol was not explicit this caused confusion and frustration:

*I was a little frustrated […] there was an in-depth look […] took a long time. This seems a grey area as to whether 60 min is when we arrive or when catheterisation occurs. (R9).*

### In-hospital

#### Learning and marginal gains

This theme encapsulated the attention to detail in every aspect of the system to improve its speed and reliability. Every case was seen as an opportunity to learn for the next time. The motivation was to highlight system, rather than individual, faults. System-specific enhancements incorporated technical and non-technical factors, for example, building ramps to help transfer the ECPR trolley, and empowering catheter laboratory nurses to be scene leaders.

*We set this time at 15 min and whenever this target was breached looked for system faults that could be refined. (R17)*

#### Standardise, simplify and protocolise

##### Protocol algorithms

Clinicians reported the standardised, methodical approach to every ECPR case was beneficial. Following a familiar algorithm allowed for an efficient but thorough progression of the process from team activation (via application displaying when and where) to post-cannulation care. Having a leader read aloud the protocol to the team was thought to be beneficial:

*It’s essential that either the ECPR nurse or perfusionist reads out the shock criteria. (R15)*

##### Equipment

Having all, but only essential, equipment present in the accessible ECPR trolley was considered advantageous. This included other ergonomic considerations such as having as much equipment in a single sterile pack as possible to reduce preparation time, and concurrently developing the procedure to use a minimal number of items.

*ECMO trolley- to be able to find all equipment required in one place (R17)*

##### Technical procedure

Respondents noted the value of a familiar, drilled and deliberated procedural technique:

*During the procedure, always using same equipment, technique and steps of cannulation is key to reduce the time during cannulation. (R21)*

The procedure was carefully developed to be fast and practical. This included training with ultrasound imaging, shorter (145 cm) wires and minimal dilators, then honing the skills with synchronised collaboration of the assisting nurses in simulation.

##### Eligibility criteria

Clear and strict eligibility criteria were identified as invaluable to aiding decision-making. Clinicians reported potential emotional factors that could complicate these decisions. However, it was noted that the senior decision-maker (consultant doctor) could be uneasy with the steep change from careful consideration to a tick-box mentality for such a consequential decision.

*Screening the patients using very specific algorithms helps to reduce any human factors. (R21)*

#### Training and simulation

The importance of having a cross-discipline training programme which incorporated regular simulations was a common response. It was noted that practising with the resuscitation and pre-hospital teams was useful in maintaining skills and coordination throughout the system. The difficulty, but necessity, of sufficient training for a HALO event was acknowledged.

*Simulations for all parties involved to help with practicalities of cannulation. (R17)*

#### Nurturing an effective team

##### Leadership- clear instruction and decision-making

Respondents perceived the need for a clear leader who was responsible for coordinating the scene by closely following and announcing the protocol: “*gave clear and precise instructions.” (R16).*

However, leadership emerged as more complicated because often the most appropriate person to coordinate the scene was someone other than the most senior doctor, whose role was cannulation and eligibility decision-making:

*Team leadership is complex, the cannulators should not also be running the code! (R19)*

##### Establishing team roles

An ECPR team can comprise staff from a wide multidisciplinary background and establishing clear roles was commonly identified as beneficial for efficiency and communication: “*Everyone knows their roles in the team (R18).”* Those without a role contributed to overcrowding which was counterproductive.

##### Balance experience and staffing

This sub-theme captured the trade-off between having a small, experienced, cohesive team and being able to staff the system sufficiently. As the size of the team increases, the number of opportunities to maintain skills decreases. Inexperience and unfamiliarity were identified as hindering the process: “*Inexperienced staff can find it very overwhelming.” (R20).*

Meanwhile, scarce appropriate staff was also highlighted: “*One of the biggest challenges is to ensure the team is available 24/7.” (R21).*

##### Welfare

Clinicians expressed the emotional burden that accompanies these high-pressured and high-stakes cases. When feasible, a ‘hot’ debrief and time were valued before resuming responsibilities. Respondents acknowledged the need for psychological support:

*It can be traumatising and our staff psychology team are helpful with after-action reviews, debriefs and counselling sessions. (R17)*

## Discussion

This study is the first to appraise the perspectives of clinicians directly involved in an existing in-hospital ECPR system for OHCA. It aimed to identify key barriers and considerations needed during the implementation of a successful ECPR system that addresses complex logistical and temporal demands. To achieve this, we analysed survey responses collected from both in-hospital and pre-hospital TVAA critical care clinicians involved in ECPR cases at a single centre. This study, while not exhaustive nor comprehensive, provides a more holistic understanding of the current regional practice and experience gained in developing a new ECPR programme which may be valuable for other services seeking to establish similar ECPR systems.

The clinicians' responses suggest that several interconnected aspects could act as barriers or facilitators within the pre-hospital and hospital phases. These included the need for inter-service communication and concurrent activity, which was central to coordinating multiple independent systems to seamlessly execute several joined up care processes quickly and safely. Furthermore, systems designed to facilitate and acknowledge human factors were key, and include clear, standardised and simplified protocols to aid decision-making and cognitive load. To build such systems, a learning culture and focus on marginal gains were vital to refine and simplify processes. Extensive education and training were crucial to equip individuals with the required knowledge and skills to perform their roles. Non-technical aspects were focused on building a highly functioning team across disciplines and services. Important contributors to this teamwork included clear leadership, established team roles, and a collective team-based approach. Here we will discuss some of the pertinent findings in relation to the literature.

Disseminating awareness to ambulance crews, who were from separate organisations, is a significant challenge shared with other ECPR systems [45, 55, 56]. Prioritising the education and training burden for new ECPR protocols, applicable to a relatively small patient group, may be difficult to justify when compared to the extensive scope of the ambulance service workload. Although the present ECPR protocol does not require ambulance crews to train to manage ongoing resuscitation efforts on route or accept responsibility for the selection and transport of appropriately filtered patients, an awareness of the program was essential to allow a shared understanding and acceptance of the reasons for deviating from the longstanding practice of resuscitation on-scene. Moreover, disseminating awareness can be complex because ambulance crews can be external to the local ambulance service (private providers, out-of-area calls). Creative solutions such as ECPR stickers on defibrillators could be utilised to develop awareness [55].

In our study, clinicians strongly advocated for standardisation and simplification of the system. An expert consensus identified 101 essential items that hospital teams must complete for OHCA ECPR [57], and performing most of these items within the 15 min allocated was an impressive feat requiring succinct processes. Of these items, clinicians have previously identified the processes of decision-making and dilation (procedure to obtain large-bore vascular access) as time-consuming and challenging [44]. Although different methods were used in our study, respondents infrequently reported these aspects as barriers, except when procedures were not followed or had not yet been implemented. Conversely and encouragingly, our results suggested consultant-led decisions based on simple and binary inclusion criteria and a standardised technical procedure, were instead facilitators. The procedure was developed to include minimal dilators and clear, drilled roles for nurses and doctors to complete tasks concurrently, reducing time and resource wastage. Furthermore, the impact of implementing strict protocols has been evidenced in a pre-hospital ECPR system [46], with time decreasing and outcomes improving markedly.

While protocols can guide clinicians, our results emphasised the importance of empowering pre-hospital critical care teams to use judgement, not just algorithms, to complete a task. This concept is known as mission command in the military [58], and was particularly valuable to allow flexibility during ALS management in transport to account for patient and clinician factors.

Another aspect identified in both pre-hospital and hospital phases was the value of simulation training. After implementing simulation training, an Australian centre observed a median decrease from 87 to 70 min from OHCA to ECPR initiation, although this was confounded by integrating a structured protocol simultaneously [59]. Studies further support using simulations to optimise team performance, enhance technical ability, and identify logistical and system deficits [25, 56, 60].

Our results align with other research advocating the pivotal role of human factors in integrating ECPR systems [60]. While concepts such as communication, leadership and teamwork are difficult to factor for directly, multi-disciplinary simulations are associated with improvements in these domains [61]. It has been established that ECPR systems require a leader to manage the scene, coordinate progression and ensure the delegation of clear roles [25, 60]. Our findings suggest this leader should not be the senior cannulating doctor, despite often being the decision-maker. Team members found clear instructions and protocol announcement facilitating- an improbable task for a task-focused cannulator to perform simultaneously*.* The concept of a ‘hands-off’ coordinator, not assumed from seniority, is widely accepted in trauma and resuscitation teams and might require concerted integration into ECPR systems [62, 63]. Furthermore, our findings emphasised the importance of shared goals in multi-disciplinary team performance, similar to their crucial role in successful, diverse sports teams [64]. Educating members about the potential benefits of ECPR further fostered this collective effort.

Multiple studies note the value of early stakeholder involvement in developing ECPR systems [45, 56, 65]. This involvement is part of a broader learning process to make iterative improvements. Marginal gains is a concept embedded in elite sporting systems such as Formula One, and is applicable to already high-performing healthcare systems [66, 67]. In the presented system, the concept acknowledges a single radical adjustment is unlikely to cut minutes from an ECPR case. Instead, many small alterations can, and do, cumulate to outcome-changing time savings [33]. This effect was observed in the evaluation of a pre-hospital Parisian system development whereby analysis concluded the culmination of many changes, rather than one identifiable area had substantially improved speed and patient outcomes [46]. To facilitate this learning and development, individuals need the opportunity to report errors and a culture that values feedback rather than issuing blame. [60].

In concordance with our findings, a major challenge for any ECPR system is the compromise between staffing a system 24/7 and the reciprocal decline in an individual’s practice frequency [56, 68]. A highly experienced, small team has been identified as one contributor to the superiority of the Minneapolis system compared to other clinical trials [69]. Our study found clinicians’ experience invaluable, but the challenge remains when serving a lower-volume population.

Excluding standardisation, our study did not identify post-cannulation hurdles as reported elsewhere. These include the provision of nursing care [70], end-of-life decision-making [41], and optimal management strategies [71]. We suspect the wording of open questions inadvertently steered responses towards the arrest to cannulation period and, as such, could be revised in future research.

The present service using pre-established critical care teams rather than local ambulance crews has benefits and drawbacks. Guidelines dictate that resuscitation quality should not be compromised to incorporate ECPR [14]. The ability of road ambulance crews to safely deliver CPR during transport has been questioned [72, 73], although the use of mechanical devices can support consistent high-quality CPR in this scenario [73, 74]. Despite OHCA being common [1], the breadth of ambulance crew work and number of personnel results in individual clinicians attending minimal cases, which limits clinical exposure and experience levels. Additionally, ECPR eligibility is rare with approximately only 10% of OHCAs meeting inclusion criteria [75, 76]. It would therefore be uncommon for ambulance crews in SCAS (where this study and ECPR programme was conducted) to perform ALS during transfer, but this is a more regular practice for TVAA critical care practitioners in the region, whose skillset is developed for HALO procedures requiring extensive training and experience along with the ability to intubate and secure an airway for improved ventilation whilst on-route to ECPR [77]*.* This frequency may explain why our results suggested resuscitation quality was likely not compromised, but in other ECPR systems low end-tidal-CO_2_ (indicative of either poor resuscitation, ventilation or physiologically inappropriate patients) [78] was identified as a major and modifiable reason to not cannulate upon hospital arrival [79]. However, one compromise is that critical care teams must often travel further to the scene, risking increased low-flow times which can equally preclude cannulation.

ECPR systems face many strategic-level challenges in addition to the functional aspects discussed here. The aim of providing 24/7 equitable provision within the time requirements is likely to require a regionalised and mixed-system approach, rather than attempting to apply this system universally.

Key recommendations from this study are found in Table 3.

**Table 3:** Key recommendations for implementing an ECPR system for OHCA

Key recommendations
Prioritise education and awareness of relevant ambulance services, including justification for ECPR. Allow time and utilise a variety of dissemination methods
Perform regular high-fidelity simulations with all teams involved (pre-hospital, ECPR, resuscitation), focusing on technical aspects and human factors, and learn from them
Learn from every case. Aim to identify any and all system-specific barriers and implement systems to overcome these where feasible
Empower critical care specialists to adapt management of patients as they see appropriate for a given situation (mission command)
Utilise critical care specialists at the operations centre to support teams and advise ambulance crews
Employ a simple pre-alert, without need to seek acceptance. This could include a direct escalation via switchboard, and a short clinical conversation with on-call consultant to aid decisions whether to open sterile equipment
Standardise handover in terms of information given and develop a hospital culture to be engaged and listen only once, within the cardiac catheter lab
Develop clear, unambiguous patient inclusion criteria. Consider the information present and reassess when new information presents
Protocolise, simplify and adapt every process including escalation, the technical procedure, and the equipment used
Establish clear team roles including a non-cannulating scene leader responsible for protocol adherence
Read the protocol aloud to the hospital cannulating team
Train as small a team as possible to feasibly staff the hospital system to facilitate maximal exposure to technical procedures
Ensure psychological support is available to team members. ‘Hot’ debrief where possible and allow time before returning to duties

Limitations and future work:

This study is limited by the inevitably small number of cases and clinicians from only one system. The limited responses precluded analysis of opinion trends stratified by clinician role or experience*.* Further to that, the snowball sampling method applied and the uncertainty around final sample sizes of in hospital teams compared to those eligible to participate limited the ability for sample size analysis or statistical powering of the study. Second, the cross-sectional design may render responses susceptible to recall bias and might fail to account for evolving viewpoints during implementation. Third, clinicians’ opinions are not analysed in the context of patient outcomes or time, so clinician-determined beneficial and detrimental factors are not validated. Fourth, although we targeted a multidisciplinary clinical cohort, the survey excluded other stakeholders including ambulance personnel, dispatchers, organisation leadership, ethical and economic decision-makers, and- crucially- patients and families [80]. Finally, the wording of the questions may have constrained, rather than guided, the responses provided.

Considering the limitations discussed, future work should be undertaken to expand the findings of this survey and to compare its relevance to other organisations and clinicians’ perspectives of similar services with different systems. Further studies with greater numbers are also needed in order to expand the certainty of findings drawn from this study. Qualitative interviews or focus groups would prove helpful to further the work done within this study and uncover a more nuanced and targeted understanding with greater clinical certainty.

## Conclusion

ECPR systems can improve outcomes in a selected cohort of refractory OHCA. This study evaluated clinicians' perspectives on how an ECPR system for OHCA can fulfil demanding criteria. The study revealed multiple interconnected technical and human factors that clinicians perceived as influential. The results presented provide important considerations for centres seeking to establish a similar service, although systems must be adapted to meet distinct regional needs.

## Supplementary Information

Additional file 1.

## Data Availability

No datasets were generated or analysed during the current study.

## Abbreviations

OHCA: Out-of-hospital cardiac arrest
CPR: Cardiopulmonary resuscitation
ECPR: Extracorporeal cardiopulmonary resuscitation
ECMO: Extracorporeal membrane oxygenation
HALO: High acuity, low occurrence
TVAA: Thames Valley Air Ambulance
ALS: Advanced Life Support
