# Supplementary material for: Clinicians’ experience of barriers and facilitators to care delivery of an extracorporeal cardiopulmonary resuscitation service for out-of-hospital cardiac arrest: a qualitative survey
Source: Scand J Trauma Resusc Emerg Med. 2024 Sep 13;32:86. doi: 10.1186/s13049-024-01261-7 (PMC11401370; doi:10.1186/s13049-024-01261-7)
Supplement: Supplementary file 1 — Additional file 1. [file 13049_2024_1261_MOESM1_ESM.docx]

**Title: What are the challenges of establishing a service to provide in-hospital ECPR for out-of-hospital cardiac arrest (OHCA)? A mixed methods study surveying pre-hospital and hospital clinicians with experience of in-hospital ECPR for OHCA.**

 Estimated completion time: **12-14 minutes**, although this may depend on how many open comments you wish to share. You are able to leave the questionnaire at any point, should you wish to do so. 

 This study aims to capture the experiences and opinions of the clinicians involved in providing an in-hospital ECPR service to those with out-of-hospital cardiac arrest. Different systems of ECPR exist around the world and contribute to varying estimates of its efficacy. From this survey of clinicians involved in the recently established pathway between Thames Valley Air Ambulance and Harefield Hospital, we hope to establish the key successes and challenges of this specific system. We hope the learning points established will contribute to the development of other successful ECPR services across the UK.

 The survey will consist of three sections. In total, there are **5 free text answers** to collect in-depth information on your opinions. Please provide **as much detail as you can.** We recommend completing this survey on a laptop/computer.

 This study is being conducted by Jasper Eddison (Imperial College London, jasper.eddison23@imperial.ac.uk), Dr James Raitt (TVAA Critical Care Consultant) and Dr Alex Rosenberg (Consultant Intensivist and Harefield clinical ECMO lead).

According to the HRA decision tool, formal NHS research ethical committee approval is not necessary. Institutional approval from TVAA and RBHT has been granted.

You will be invited to submit your email address if you are happy for us to contact you to follow up. However, this is optional should you wish to remain anonymous.

All data will be securely stored by Qualtrics XM (an online survey software provided under licence by Imperial College London) before being exported to secure software such as a password-protected Microsoft Excel document in an encrypted cloud. No patient data is involved at any stage. Please do not enter any patient-identifiable data into your free-text answer.

 By submitting your answers, you consent to us collecting this data and acknowledge that data may be used for publication.

I have read the introduction and consent to my data being used in this study:

- I agree

End of Block: Introduction

Start of Block: Background Information: About You

This survey is focused on all cases of ECPR pathway activation for OHCA, regardless of cannulation or outcome.

I am a...

If Harefield Hospital Doctor, what is your base speciality?

In total, how many **out-of-hospital cardiac arrest** ECPR cases have you been involved in, regardless of whether the patient was cannulated or not?

In total, how many **in-hospital cardiac arrest** ECPR cases have you been involved in?

At what stage were you involved with the out-of-hospital ECPR case (tick all that apply)

In your own words, what was your role during the case?

________________________________________________________________

End of Block: Background Information: About You

Start of Block: Block 2

The following questions involve free-text answers to give you the flexibility to highlight key aspects of the service. Please give as many examples as you can and provide appropriate explanations of why these were successes/ challenges where possible.

In the case(s) you have been involved in, please explain the **technical aspects** (equipment/ procedural/ systems) of the case that **went well/ contributed positively/ were helpful**.

________________________________________________________________

In the case(s) you have been involved in, please explain the **non-technical aspects** (human factors, crew resource factors, teamwork, leadership, decision-making) of the case that **went well/ contributed positively/ were helpful**.

________________________________________________________________

In the case(s) you have been involved in, please explain the **technical aspects**(equipment/ procedural/ systems) of the case that **were challenging/ were a barrier/ had scope for improvement**.

________________________________________________________________

In the case (s) you have been involved in, please explain the **non-technical aspects** (human factors, crew resource factors, teamwork, leadership, decision making) of the case that **were challenging/ were a barrier/ had scope for improvement.**

________________________________________________________________

Please rate how you feel in regard to the following statements

| Not applicable to me | Strongly disagree | Somewhat disagree | Neither agree nor disagree | Somewhat agree | Strongly agree |
| --- | --- | --- | --- | --- | --- |

|  |
| --- |
| I feel confident identifying inclusion and exclusion criteria in patients. |
| I feel compelled to break inclusion/ exclusion criteria in certain patient groups. |
| I feel confident to use my own clinical judgement and experience to make decisions outside of the standard operating procedure. |
| I feel confident that communication with other emergency services does not delay patient transfer. |
| I feel confident in providing high-quality ALS care during transport. |
| I feel that the hospital team is adequately prepared when the patient arrives. |
| I feel this service provides the opportunity for me to develop professionally. |
| I feel supported by my team and organisation to manage the emotional burden that can accompany these cases. |

Please rate how you feel in regard to the following statements

| Not applicable to me | Strongly disagree | Somewhat disagree | Neither agree nor disagree | Somewhat agree | Strongly agree |
| --- | --- | --- | --- | --- | --- |

|  |
| --- |
| I feel that the hospital team is adequately prepared when the patient arrives. |
| I feel confident applying the STOP criteria upon my primary assessment of the patient. |
| I feel compelled to break inclusion/ exclusion criteria in certain patient groups. |
| I feel confident using the equipment during cannulation. |
| I feel the frequency of cannulation (simulation or clinical) has maintained my competency. |
| I feel that the environment used for cannulation is appropriate. |
| I feel confident about my specific role during an ECPR call. |
| I feel this service provides the opportunity for me to develop professionally. |
| I feel supported by my team and organisation to manage the emotional burden that can accompany these cases. |

Please rate how you feel in regard to the following statements:

| Not applicable to me | Strongly disagree | Somewhat disagree | Neither agree nor disagree | Somewhat agree | Strongly agree |
| --- | --- | --- | --- | --- | --- |

|  |
| --- |
| I feel that the ECPR service puts a disproportionate strain on intensive care resources. |
| I feel the intensive care team has enough experience to manage this complex patient cohort. |
| I feel there are sufficient appropriately trained ECMO nurses to provide this service. |
| I feel there is a high enough frequency of ECMO/ECPR cases to maintain clinical competency across the intensive care team. |
| I feel there is adequate guidance regarding end-of-life care decisions. |
| I feel this service provides the opportunity for me to develop professionally. |
| I feel supported by my team and organisation to manage the emotional burden that can accompany these cases. |

Any additional comments

________________________________________________________________

End of Block: Block 2

Start of Block: Block 3

Based on your experience of ECPR for out-of-hospital cardiac arrest, what key advice would you give to counterparts looking to set up an equivalent service?

________________________________________________________________

Please rate how important you feel these advances are for the continuing improvement of the ECPR service.

| Unsure | Not at all important | Slightly important | Moderately important | Very important | Extremely important |
| --- | --- | --- | --- | --- | --- |

|  |
| --- |
| Further public education to increase bystander CPR. |
| Technological advancements at dispatch from initial emergency calls to identify suitable patients (e.g. using rescuer mobile video footage). |
| Enhancing rapid identification of patients and access to their medical history before cannulation. |
| Stronger evidence in the literature supporting its use. |
| Transmission of patient observations/status to the hospital team during transfer. |
| Improved clinical/ procedural skills. |
| Improved cannulation procedure (equipment and techniques). |
| Developing an organ donation system in the event of a poor outcome. |
| Greater understanding of post-resuscitation management. |

| 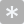 |
| --- |

Please provide your contact email below if you would be happy to receive a follow-up email. This may include an invitation to interview/ focus group or follow-up questions which could support the findings of this study.

________________________________________________________________

End of Block: Block 3
